# Forskolin and IBMX Induce Neural Transdifferentiation of MSCs Through Downregulation of the NRSF

**DOI:** 10.1038/s41598-019-39544-0

**Published:** 2019-02-27

**Authors:** Ryan Thompson, Christina Casali, Christina Chan

**Affiliations:** 1 Cell and Molecular Biology Program, Michigan State University, 567 Wilson Road, Rm 2240E, East Lansing, Michigan 48824 USA; 2 Department of Chemical Engineering and Materials Science, Michigan State University, 428S. Shaw Lane, Rm 2527, East Lansing, Michigan 48824 USA; 3 Department of Biochemistry and Molecular Biology, Michigan State University, 603 Wilson Road, East Lansing, MI 48824 USA

## Abstract

Neural differentiation of mesenchymal stem cells is a controversial phenomenon, as it would require transdifferentiation across the mesoderm-ectoderm barrier. However, several laboratories have observed that MSCs are able to be induced to express neural characteristics. Previously, we demonstrated that the cAMP-elevating agents, forskolin and IBMX, induced neural-like differentiation of MSCs, including expression of neural markers and increased sensitivity to neurotransmitters. However, due to the broad range of effects that forskolin and IBMX can elicit through the intracellular second messenger, cAMP, a better mechanistic understanding is required. Here, we show that neural induction by forskolin and IBMX is dependent on downregulation of expression of the master transcriptional regulator, neuron restrictive silencer factor (NRSF), and its downstream target genes. Since silencing of NRSF is known to initiate neural differentiation, it suggests that forskolin and IBMX result in transdifferentiation of MSCs into a neural lineage.

## Introduction

Mesenchymal stem cells (MSCs) are multipotent adult stem cells that constitute an important part of the bone marrow microenvironment providing cell-cell contacts and secretion of trophic factors needed to support the growth and development of various resident cell types. Additionally, as a stem cell, MSCs serve as the progenitor for the osteogenic, chondrogenic, and adipocytic lineages^1^. Because of their ease of attainment from bone marrow and adipose tissue^1–3^ and their high rate of proliferation, MSCs have been a convenient stem cell type for *in vitro* study. In particular, research into their highly plastic nature has revealed that MSCs can be induced to differentiate beyond their canonical lineages into renal, hepatocytic, cardiac, pancreatic, and neural cells^4–7^. The prospect of generating large amounts of cell types from MSCs could have important therapeutic implications. MSCs are an attractive candidate for cell replacement therapies from a therapeutic perspective, considering their potential for autologous grafting and their low risk of tumor formation post transplantation^8,9^.

Among the pathologies that could benefit from cell replacement therapies, neurodegenerative diseases including Parkinson’s Disease and Alzheimer’s Disease are self-evident. Not surprisingly, this has driven much research into inducing neural differentiation of MSCs, with the principal goal of generating specific neural functions. *In vitro* experiments have shown that MSCs can be induced to gain characteristics of neural cells including spontaneous generation of Na+/K+ currents, expression of neural specific structural proteins, and exhibition of neuronal morphology^10–15^. Additionally, MSCs can be induced to express key neural genes involved in the synthesis and transmission of neurotransmitters, chief among them, the rate-limiting enzyme of dopamine synthesis, tyrosine hydroxylase (TH).

Neural differentiation of MSCs remains a controversial topic because it requires transdifferentiation across the mesoderm-ectoderm germline barrier. Despite acquisition of neural functions, several studies have questioned the extent to which MSCs can “differentiate” into neurons^16–19^. In order to justify the expression of neural characteristics induced in MSCs, better characterization of the molecular mechanisms driving differentiation is needed.

Previously, our laboratory showed that a combination of forskolin and IBMX (FI), could induce neural differentiation of MSCs. Changes included expression of neural markers, a change in cell morphology, and increased sensitivity to the neurotransmitter, dopamine^10^. Forskolin and IBMX are small molecules that elevate the intracellular concentration of the second messenger, cyclic adenosine monophosphate (cAMP). While cAMP is known to play a role in neural differentiation^20–22^, how it induces differentiation of MSCs is unclear. Rises in intracellular levels of cAMP signal through protein kinases to activate the transcription factor CREB. However, CREB is highly pleiotropic and is involved in the development of tissues derived from the endoderm, ectoderm, and mesoderm. A better characterization of the mechanism is needed to explain the neural-inducing effect of FI within the mesodermal background of MSCs.

Transcription factors are critical for specifying cell lineage. Indeed, reprogramming cells with forced expression of transcription factors can transdifferentiate cells across the germ line barrier^23–25^. To better understand neural induction of MSCs with FI we asked if FI could be affecting neural-specific transcription factors. Previously, Yang *et al*. demonstrated that knockdown of the master transcriptional repressor of the neural phenotype, the neuron restrictive silencer factor (NRSF), induces neural gene expression, gain of neuronal morphology, and causes the cells to generate spontaneous action potentials^26^. NRSF is a transcriptional repressor that is ubiquitously expressed in NSCs as well as in non-neural tissue. NRSF binds to a conserved 21-bp neuron restrictive silencer element that is often found on the promoters of neural genes where it then recruits histone deacetlyases and DNA methylases to repress gene expression^27^. Silencing of NRSF alone results in MSCs that spontaneously fire Na+ currents, a distinct gain of neuronal morphology, and expression of a variety of neural genes including BDNF and NSE^26^. Because of the importance of NRSF in neural differentiation, we questioned whether FI-induced differentiation affects NRSF expression to promote neural differentiation in MSCs.

Given that both FI as well as knockdown of NRSF in MSCs cause neural differentiation, we hypothesized that FI had a regulatory effect on NRSF. We report that FI downregulates expression of NRSF and that this event is responsible for the expression of neuronal genes and for the increase in sensitivity to neurotransmitters in MSCs. Knockdown of NRSF recapitulates the changes observed during FI induced differentiation and overexpression of NRSF is able to block expression of neuronal genes in FI-treated MSCs. We propose that the mechanism behind FI induced neural transdifferentiation of MSCs requires the downregulation of NRSF.

## Materials and Methods

MSCs were isolated from animals using procedures approved by the Institutional Animal Care and Use Committee at Michigan State University. All recombinant DNA used in experiments has been registered with the Institutional Biosafety Committee at Michigan State University. All experiments and methods were performed in accordance within the relevant guidelines and regulations of the Biological Safety and Chemical Hygiene plans set for by Environmental Health and Safety Department at Michigan State University.

### Materials

For detailed information on materials, antibodies, and primer sequences see Supplemental Tables 1–3 in the Supplemental file.

### Mesenchymal stem cell culture and isolation

MSCs were derived from bone marrow isolated from 4 to 6 week-old Sprague-Dawley female rat as previously described^10^. Femurs and tibias were removed from 4 to 6-week-old rats. The two ends of the bone were cut open and the marrow was flushed with 10 mL of DMEM using a 25 g needle and syringe. The cell suspension was filtered through a 70-um nylon mesh to remove bone debris and blood aggregates. Cells were cultured in low glucose DMEM (Invitrogen) supplemented with 10% fetal bovine serum (Invitrogen) and free from antibiotics. Cells were incubated in a humidified atmosphere containing 5% CO2 at 37 C. Non-adherent cells from the flushed marrow were removed after 48 h after isolation. Media was replaced every 3 days until the cells reached 80–90% confluence. Confluent cells were detached by 0.25% trypsin–EDTA (Invitrogen) and plated for further experiments. Neural differentiation was induced by culturing MSCs in the presence of growth media supplemented with 10 uM forskolin (Sigma) and 100 uM isobutylmethylxanthine (IBMX) (Sigma) for up to 5 days.

### Cell Transformation

Plasmid complexes were prepared in Opti-MEM (Gibco) with 1 ug of plasmid, 1 uL of P3000 reagent, and 1 uL of Lipofectamine 3000 (Invitrogen) per sample. 10^6 cells/mL were reverse transfected with prepared plasmid complexes growth medium free of antibiotics. After 16 h, the medium was replaced and the cells were grown for another 24 h before sample collection or treatment. For silencing experiments, siRNA was complexed with Lipofectamine 3000 in Opti-MEM. 10^6 cells/mL were reverse transfected with siRNA (0–50 nM) for 16 h. Afterwards, medium was refreshed and the cells were cultured for an additional 48 h before sample collection or assays.

### Western Blotting

Whole-cell extracts were prepared by lysing cells with RIPA buffer (50 mM Tris pH 8.0, 150 mM NaCl, 1% IGEPAL (NP-40), 0.1% sodium dodecyl sulfate, 0.5% sodium deoxycholate) on ice for 30 min. Nuclear fractions were prepared by swelling cells in a hypotonic buffer (10 mM HEPES (pH 8.0), 1.5 mM MgCl2, 10 mM KCl) on ice then lysing with a dounce homogenizer. The nuclei were spun down and incubated on ice in a high salt buffer (20 mM HEPES pH 8.0, 1.5 mM MgCl2, 420 mM NaCl, 25% glycerol) to extract the protein. Lysates were mixed with 5X SDS protein loading buffer (50 mM Tris pH 7.0, 25% glycerol, 2% SDS, 0.025% bromophenol blue) and denatured at 95 C for 5 min. 20 ug of each sample lysate was separated by electrophoresis on an 8% Tris–HCl gel and transferred to a nitrocellulose membrane. Membranes were then blocked in 5% milk and 0.05% Tween 20–TBS (Tris buffered saline) for 1 h and incubated with primary antibodies against tyrosine hydroxylase or GAPDH (Cell Signaling) or NRSF/REST (Millipore) overnight at 4 C. Anti-mouse or anti-rabbit HRP-conjugated secondary antibody (Thermo Scientific) was added the second day after primary antibody incubation. The blots were incubated for 90 min and then washed three times with 0.05% Tween 20–TBS. The blots were then visualized by SuperSignal west femto maximum sensitivity substrate (Thermo Scientific). Image of full blots are included in the supplemental figures section.

### Real Time PCR

mRNA samples were prepared with the RNeasy Mini Kit (Qiagen). mRNA was then reverse transcribed to cDNA using the High Capacity cDNA Reverse Transcription Kit (Applied Biosystems). Real Time PCR was used to quantify gene expression for *Th*, *Tuj1*, *Nse*, *Drd1*, *Drd5*, *Nurr1, Vmat2, and Lmx1a*. cDNA from samples was mixed with iQ Sybr Green Supermix (BioRad) and run on a MyIQ single detection Thermal Cycler. Data was transformed using the KKct method.

### NRSF Subcloning

pHR’-NRSF-CITE-GFP was a gift from Jay Nadeau (Addgene plasmid # 21310). NRSF was cloned out using PCR and the resulting fragment was cloned into a pCMV-Myc-N plasmid (Clonetech). Overexpression of NRSF was confirmed with western blotting against NRSF as well as the myc tag (Supplemental Fig. 1).

### Calcium imaging

Calcium imaging was performed according to the protocol by Tropel *et al*.^11^ and modified by Zhang^10^. Cells were cultured in four-well chambered cover-glass (Lab-Tek) coated with poly-L-lysine (Cultrex). After neural induction with FI or transformation with lipocomplexes, the cells were stained with 4 uM Fluo-4 (Invitrogen) in ACSF–HEPES (artificial cerebral spinal fluid with HEPES: 119 mM NaCl, 2.5 mM KCl, 1.3 mM MgCl2, 2.5 mM CaCl2, 1 mM NaH2PO4, 26.2 mM NaHCO3, 11 mM dextrose, 10 mM HEPES, pH = 7.4) for 30 min at 37 C. Excess dye was removed by washing cells with PBS twice and placing into a 37 C chamber on the stage of Olympus FluoView 1000. Then, 0.5 ml ACSF–HEPES was added to the well to begin imaging. Images were captured every 1.137 s and fluorescence intensity is represented by a spectral table (warmer colors represent higher intensity whereas cooler colors represent lower intensity). After 15–20 images, 0.5 ml ACSF–HEPES buffer containing the following neurotransmitters were added: 200 uM glutamate (final concentration 100 uM), 200 uM dopamine (final concentration 100 uM), or 200 uM ATP (final concentration 100 uM). A total of 200–300 images were recorded and the data was analyzed by the FluoView 100 software. Changes in the fluorescence intensity of the Ca2+ signal are represented as F/F0. The percent of responsive cells is calculated as the number of cells with a F/F0 signal greater than 20% of the total number of cells.

### Statistical Analysis

Gene expression data were determined as statistically significant by Tukey’s Test following one way ANOVA for groups with multiple means. For experiments comparing two samples a student’s *t-*test was employed. Results were presented as the average of the data set+/– the SEM (standard error of the mean). Similarly, statistical significance of calcium release quantification is also represented as the average of the data set+/– the SEM.

## Results

### FI Causes Downregulation of NRSF in MSCs

**Figure 1:**
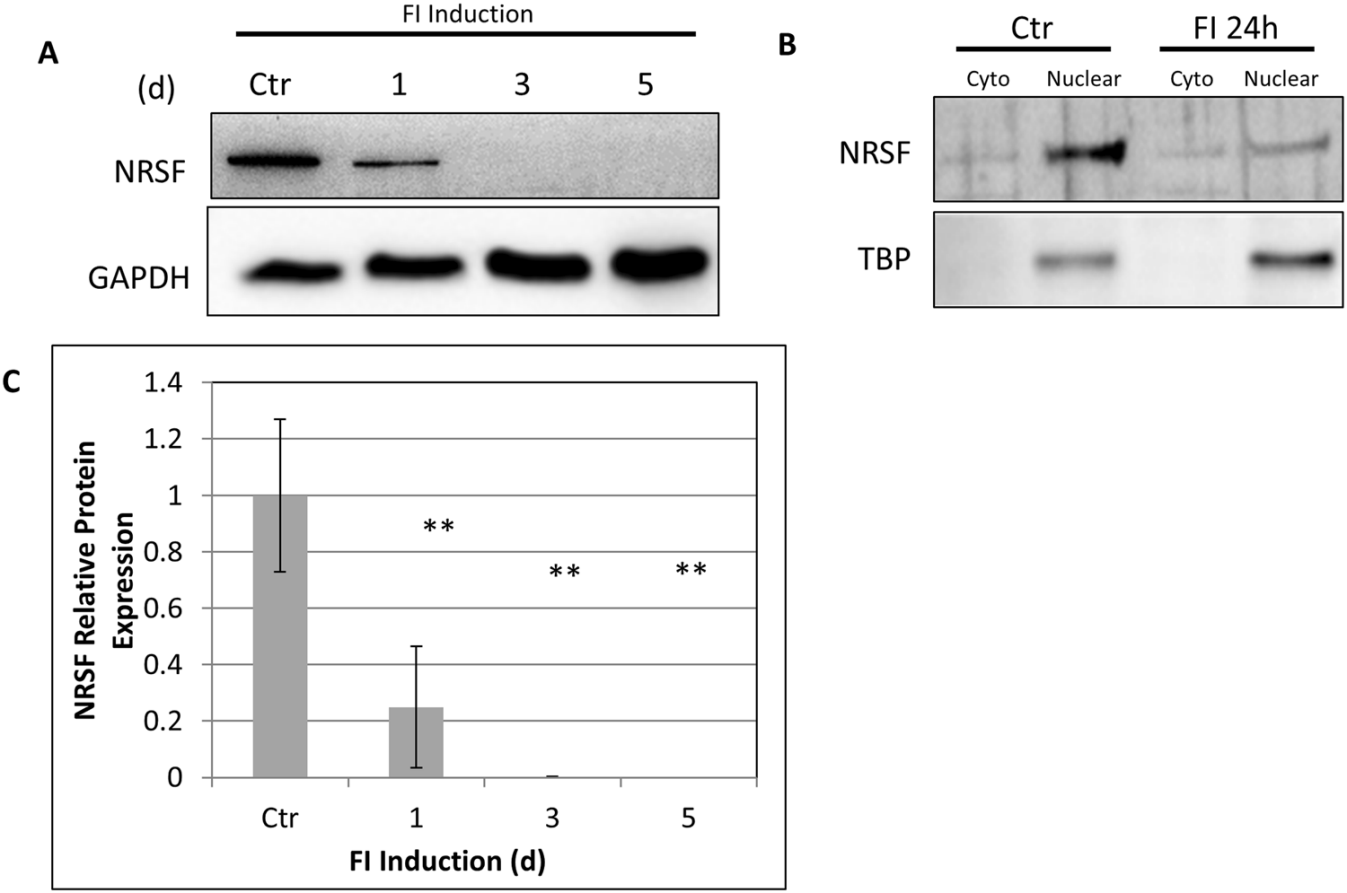
FI downregulates NRSF protein in MSCs. (**A**) Treatment of MSCs with FI over 5 days shows a marked reduction in NRSF protein expression. Full length blots are included in Supplementary Fig. 2. (**B**) This effect occurs within 24 h. (**C**) NRSF is also reduced in the nuclear fraction. **p < 0.01 as compared to control using Tukey’s test following one-way ANOVA with N = 3.

We found that inducing neural differentiation in MSCs with FI strongly downregulated NRSF protein expression after 24 h, which continued over the 5 day treatment course (Fig. 1A). Concomitantly, NRSF expression in the nuclear fraction was strongly downregulated in the FI-treated MSCs as compared with the controls (Fig. 1B). Thus, FI has a strong effect on the protein expression and localization of NRSF.

### FI Treatment Induces Gene Expression of Several NRSF Repressed Target Genes

**Figure 2:**
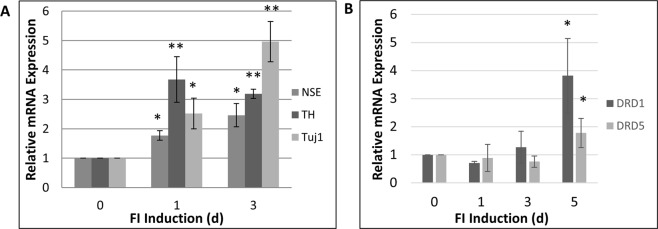
FI de-represses NRSF-dependent gene expression. (**A**) Treatment of MSCs with FI induces gene expression of several neural markers that are known targets of NRSF. (**B**) FI treatment induces expression of dopamine receptor genes. *p < 0.05; **p < 0.01 as compared to day zero control using Tukey’s test following one-way ANOVA with N = 3.

Given that FI strongly downregulates NRSF, we determined if FI caused an increase in the expression of known NRSF-repressed genes. *Tuj1* and *Nse* are well characterized genes, regulated by NRSF, that are commonly used as neural markers. Since FI-induced MSCs were previously shown to express dopamine sensitivity^10^, we assayed for tyrosine hydroxylase (*Th*) expression. *Th* is the rate-limiting enzyme for dopamine synthesis that is specific to dopamine producing neurons and is known to be repressed by NRSF^28^. The gene expression levels of *Th*, *Tuj1* and *Nse* increased 24 hours after FI treatment reflecting the corresponding decrease in NRSF protein expression. This continued through the three days of treatment (Fig. 2A), suggesting a relationship between FI and NRSF repression of gene expression. Since we previously demonstrated that FI induced dopamine sensitivity in MSCs^10^ we investigated changes in gene expression that could explain this gain of dopaminergic function. We measured the expression of dopamine receptor genes (*Drd)*. Notably FI induced expression of *Drd1* and *Drd5* (Fig. 2B). Interestingly, the dopaminergic marker, *Th*, is regulated directly by NRSF through multiple NRSEs within its promoter region^28^. Since this is a critical marker for dopaminergic neurons we asked if FI induced differentiation had intrinsic bias towards dopaminergic differentiation. We observed that FI induced expression of multiple genes involved in the development and function of dopaminergic neurons (Supplemental Fig. 3). In addition to *Th*, FI induced expression of a key neurotransmitter transporter, *Vmat2*, which is important for transport of neurotransmitters into vesicles. We also observed increased expression of *Nurr1* and *Lmx1a*, transcription factors that are important for specifying dopaminergic cell fate within the midbrain region^29^. Aside from *Th*, it is not known if NRSF directly regulates other genes important for dopaminergic neurons.

### Knockdown of NRSF with siRNA Reproduces FI-Induced Gene Expression

**Figure 3:**
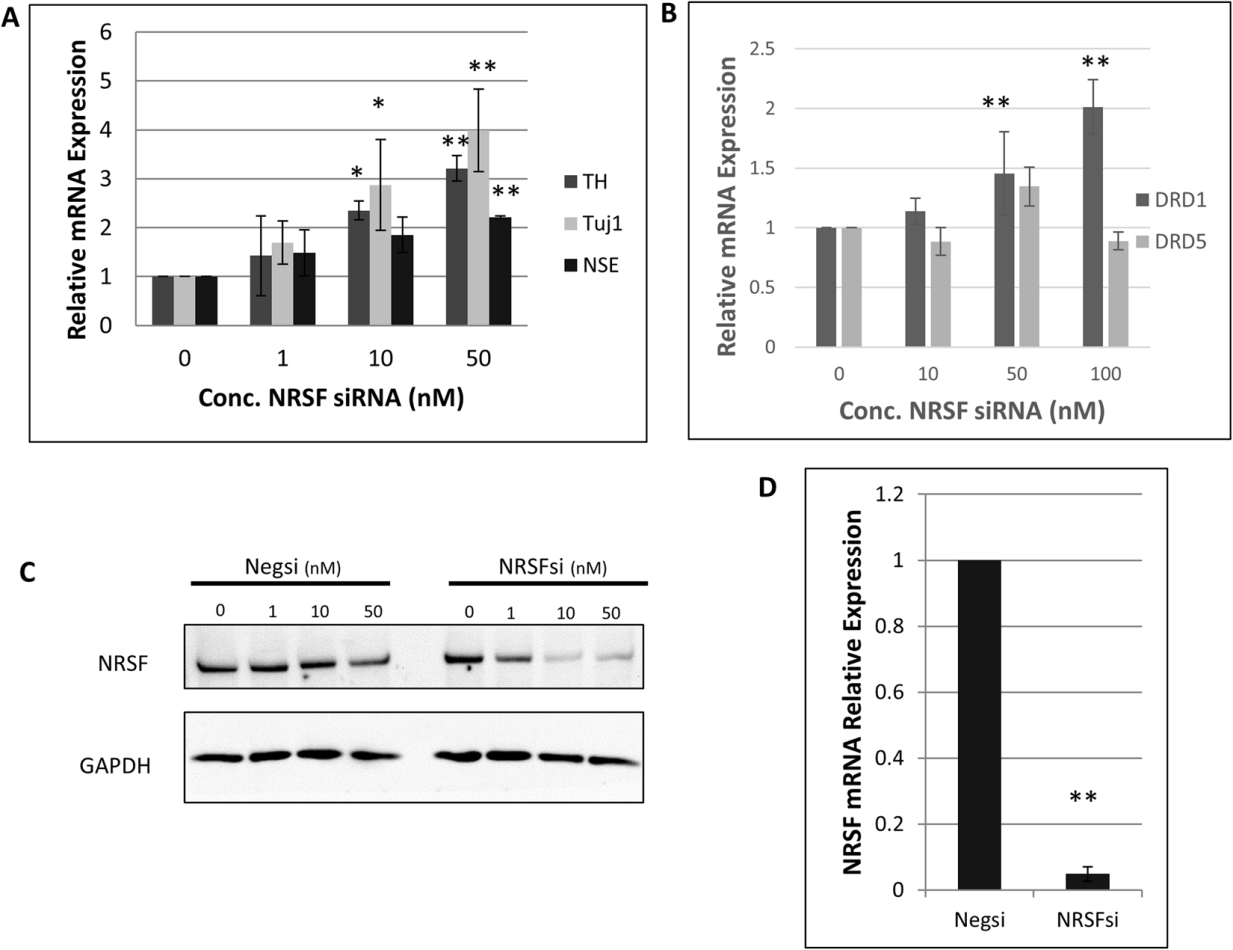
NRSF silencing de-represses FI induced genes. (**A**) Knockdown of NRSF protein using siRNA induces expression of same neural markers that FI induces, in a dose dependent manner. (**B**) Silencing of NRSF is shown to induce expression of the *drd1* receptor gene. No significant upregulation of *drd5* was observed. (**C**) Expression of NRSF protein levels with increasing concentration of siRNA. (**D**) Knockdown of NRSF mRNA expression with 50 nM NRSF siRNA. Full length blots are included in Supplementary Fig. 4. *p < 0.05; **p < 0.01 as compared to 0 nM siRNA treatment using Tukey’s test following one-way ANOVA with N = 4.

To demonstrate that the neural gene expression induced by FI in MSCs was the result of NRSF downregulation and not off-target effects of increased intracellular cAMP concentrations, we knocked down NRSF using siRNA. After silencing for 3 days we found that 10 and 50 nM of siRNA strongly downregulated NRSF protein levels (Fig. 3C) and de-repressed gene expression of *Th*, *Tuj1*, and *Nse*, (Fig. 3A). At 50 nM of siRNA, the NRSF gene expression reduced by over 90% (Fig. 3D), while 1 nM of siRNA downregulated NRSF expression, it did not significantly de-repressed *Th, Tuj1*, or *Nse* expression, suggesting that NRSF-dependent repression is dose-dependent. Another key point of our previous work was that FI induced MSCs to respond to exposure to dopamine by releasing calcium. Since FI was able to increase expression of *Drd1* and *Drd5* (Fig. 2B) we asked if silencing of NRSF could induce expression of the *Drd1* and *Drd5* genes. However, we observed that knockdown of NRSF was able to increase expression of only *Drd1* (Fig. 3B).

### Overexpression of NRSF Downregulates Target Gene Expression and Blocks FI Induced Gene Expression

**Figure 4:**
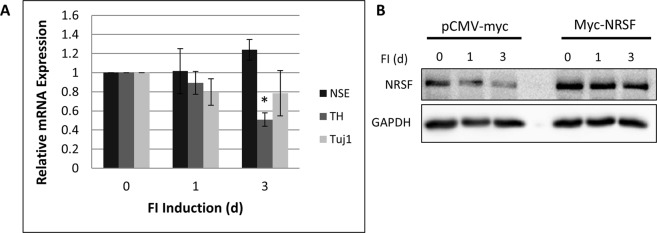
NRSF overexpression attenuates FI-induced gene expression. (**A**) MSCs were transfected with a vector overexpressing NRSF then treated with FI. Gene expression of several NRSF targets inducible by FI are no longer induced in MSCs overexpressing NRSF. (**B**) Immunoblot of NRSF expression remains elevated during the course of the FI treatment in MSCs overexpressing NRSF vs. those transformed with an empty vector. Full length blots are included in Supplementary Fig. 4. *p < 0.05 as compared with its level on day 0. Statistics were performed using Tukey’s test following one-way ANOVA with N = 3.

To determine if neural-like differentiation of MSCs was specifically the result of the downregulation of NRSF by FI, NRSF was overexpressed in MSCs by cloning murine NRSF into a pCMV myc-N-terminal vector. We overexpressed myc-NRSF in MSCs, treated the cells with FI for three days and observed that NRSF-dependent gene expression did not increase and in some cases decreased below baseline (Fig. 4A). FI treatment over the three days did not downregulate NRSF protein expression in the overexpressing cells (Fig. 4B) showing that FI induced neural gene expression is due to the de-repression of NRSF and not off-target gene activating effects of FI.

### FI-Induced Dopamine Sensitivity in MSCs is Dependent on NRSF Downregulation

**Figure 5:**
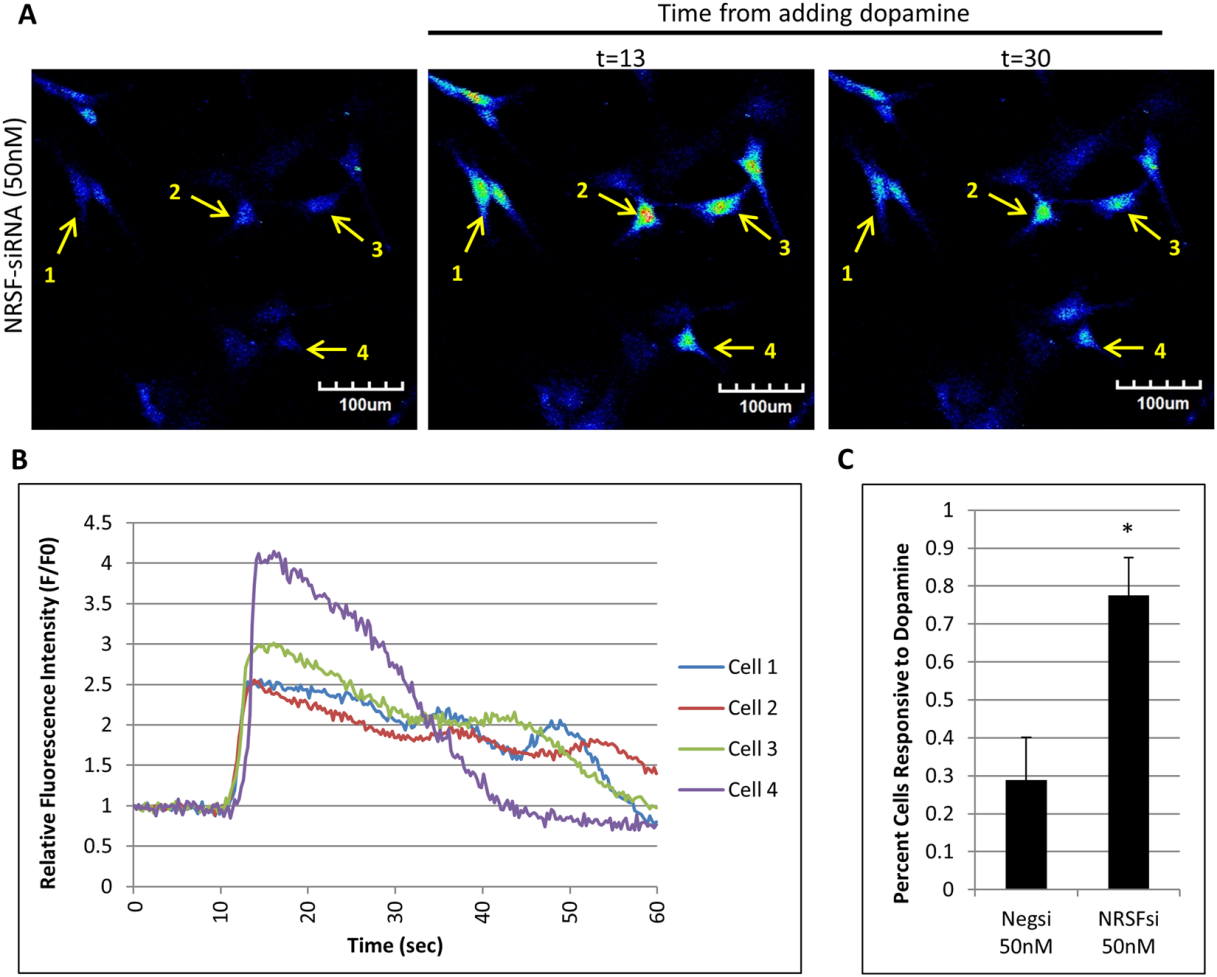
Knockdown of NRSF with siRNA Induces Dopamine Sensitivity in MSCs. (**A**) Dopamine sensitivity in MSCs silenced with 50 nM of siRNA against NRSF. Increase in fluorescence intensity represents increase in calcium release via the fluo-4 dye. (**B**) Individual fluorescence intensity of select cells over time (sec) of experiment. (**C**) Quantification of cells responsive to dopamine exposure. Increase of fluorescence intensity of 20% over initial resting fluorescence intensity (F/Fo) was counted as a positive response. *p < 0.05 using students T-test with N = 3.

As previously reported by Zhang *et al*.^10^, MSCs exhibit some sensitivity to neurotransmitters, in particular, dopamine. When exposed to dopamine, up to 80% FI-induced MSCs respond to dopamine by releasing calcium waves into their cytosol suggesting that FI induced MSCs gain neural-like signaling function. Additionally, we show that *Drd1* and *Drd5* are upregulated by FI and *Drd1* is responsive to NRSF knockdown. To determine if FI induced sensitivity to dopamine is dependent on NRSF, we used siRNA to knockdown NRSF protein expression. After 72 h the cells were stained with a calcium sensitive dye, Fluo-4, exposed to dopamine, and imaged with an Olympus Fluoview 1000 confocal microscope to observe calcium release in real time. When induced with dopamine, up to 78% of cells knocked down with NRSF siRNA became sensitive to dopamine and released calcium (Fig. 5A, Supplemental Video 1) into the cytosolic space. Cells that exhibit an increase in fluorescence intensity of 20% or greater at any point up to 60 seconds after exposure to dopamine Fig. 5B), over the fluorescence intensity at t = 0 sec were counted as sensitive to dopamine. Less than 30% of the negative control cells transfected with scrambled siRNA gained dopamine sensitivity (Fig. 5C, Supplemental Video 2). This is in agreement with our previous results^10^ showing that less than 40% of the uninduced MSCs showed dopamine sensitivity. In addition, the negative control cells that responded to dopamine showed far less intense calcium release.

**Figure 6:**
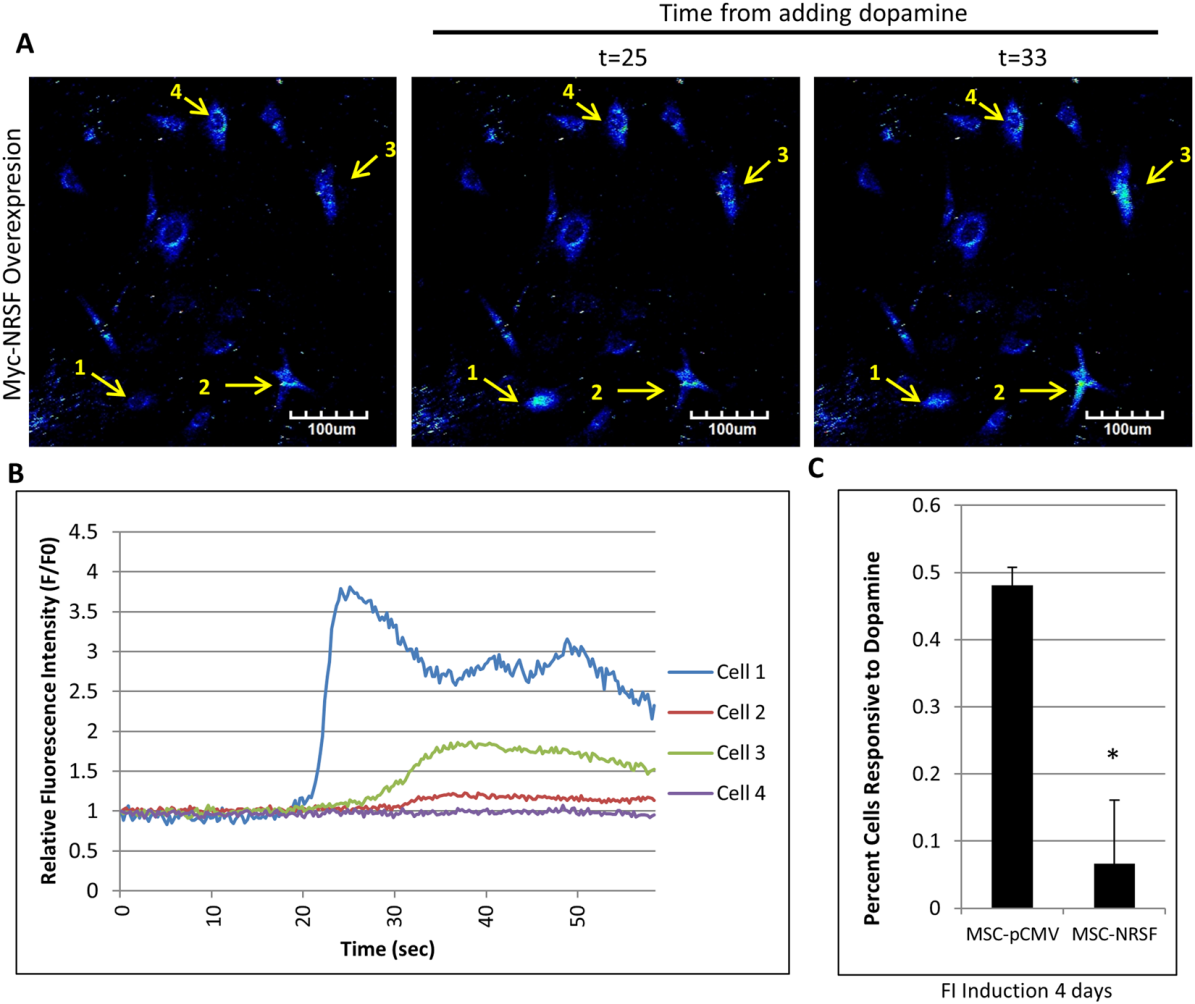
Overexpression of NRSF In MSCs Abolishes FI Induced Dopamine Sensitivity. (**A**) MSCs overexpressing NRSF from a pCMV vector are unable to respond to dopamine after being induced with FI for 4 days. Again, an increase in fluorescence intensity represents calcium release via the fluo-4 dye. (**B**) Individual fluorescence intensity of select cells over time (sec) of experiment. (**C**) Quantification of cells responsive to dopamine exposure. Increase of fluorescence intensity of 20% over initial resting fluorescence intensity (F/Fo) was counted as a positive response. *p < 0.05 using students T-test with N = 3.

To determine if NRSF inhibits MSC sensitivity to dopamine, MSCs were transfected with either pCMV-myc empty vector or pCMV-myc-NRSF and induced with FI for 4 days. MSCs overexpressing NRSF and induced with FI appeared to almost completely lose their dopamine sensitivity, with <10% of the cells responding to dopamine (Fig. 6A,B, Supplemental Video 4). In the empty vector expressing cells, FI induced an increase in dopamine sensitivity as expected with >50% of cells responding by releasing calcium (Fig. 6C, Supplemental Video 3). Taken together, FI increases dopamine sensitivity in MSCs in a NRSF-dependent manner. In addition, a small percentage of MSCs in the silencing experiments exhibited sensitivity to glutamate (Supplemental Fig. 6, Supplemental Video 5). This mirrors our previous study with FI that showed ~20% of the induced cells were sensitive to glutamate^10^.

### FI Downregulates NRSF Protein Levels through a Post-Translational Mechanism

**Figure 7:**
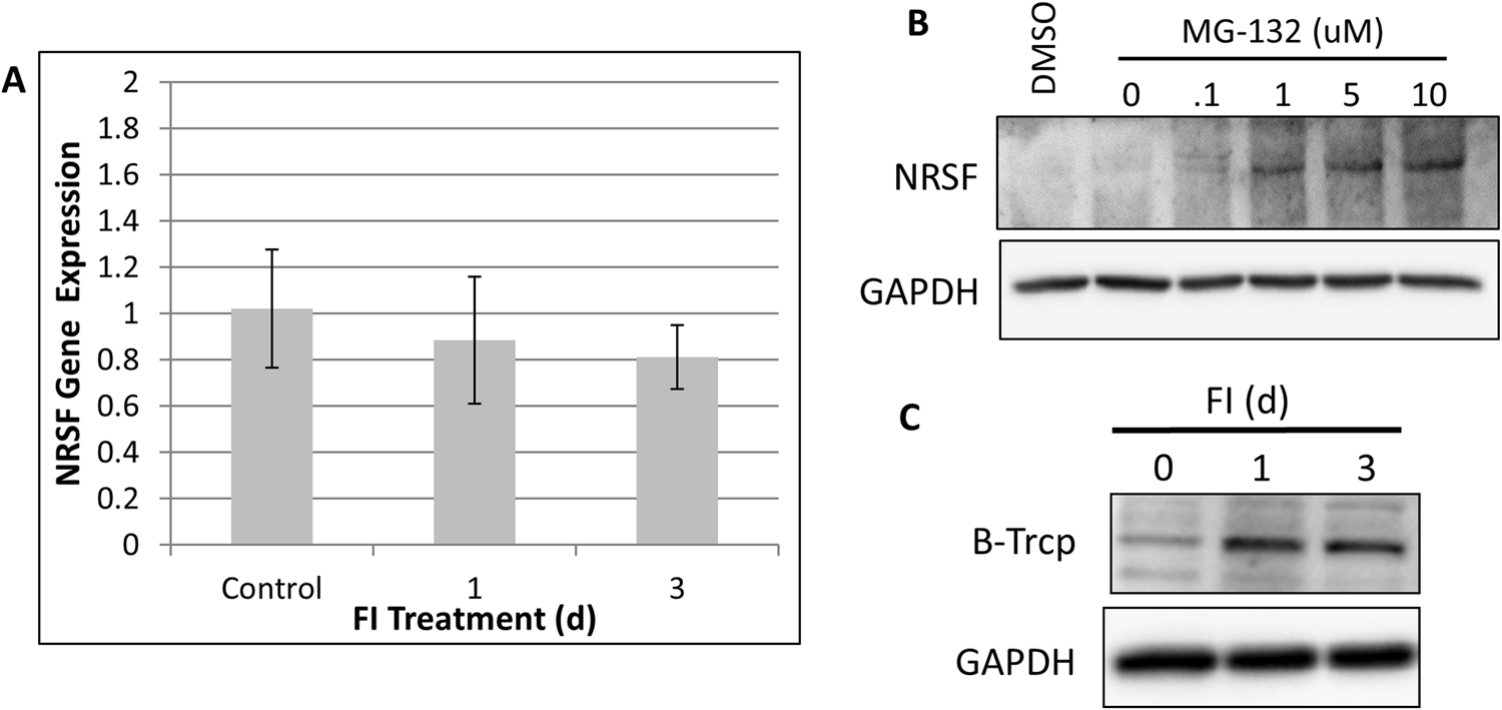
FI Induced Downregulation of NRSF involves a post-translational mechanism. (**A**) FI does not downregulate mRNA expression of NRSF despite a large decrease in protein expression. No statistically significant changes in gene expression for N = 3. (**B**) NRSF protein expression is elevated in the cells treated with proteasome inhibitor for 2 h suggesting that it is negatively regulated by the ubiquitin-proteasome system. Full length blots are included in Supplementary Fig. 6. (**C**) FI induced MSCs show increased protein expression of the E3 ligase B-Trcp.

It is not known how FI downregulates NRSF protein expression. Since FI activates the PKA-CREB signaling pathway, the downregulation of NRSF likely occurs through an indirect mechanism. MSCs induced with FI over a period of days did not significantly modulate NRSF mRNA levels (Fig. 7A), suggesting that the protein expression of NRSF is strongly regulated through a post-translational mechanism. The ubiquitin-proteasome system can downregulate protein expression by tagging target proteins with polyubiquitin chains which are recognized by the proteasome and subsequently degraded. Using the proteasome inhibitor MG-132 NRSF is strongly upregulated in the MSCs within 3 hours of treatment suggesting that it is regulated by the ubiquitin-proteasome system (Fig. 7B). This is in agreement with Westbrook *et al*.^30^ who previously demonstrated that NRSF is regulated by the E3 ubiquitin ligase B-Trcp in stem cells and cancer cells. Inducing MSCs with FI increased B-Trcp protein expression (Fig. 7C) which remained elevated during the course of the treatment suggesting that FI may downregulate NRSF protein expression by upregulating B-Trcp.

## Discussion

Previously, our laboratory reported that FI induced neural-like differentiation of MSCs and that CREB was a key transcription factor in this process^10,31^. However, due to the highly pleiotropic nature of CREB and its importance to other non-neural cell lineages, we investigated possible mechanisms induced by FI that might be specific to neural differentiation. We hypothesized that NRSF, whose expression is a major hurdle in the development and maturation of NSCs to functional mature neurons^32,33^, might also play a role in the neural differentiation of MSCs. We demonstrate here that downregulation of NRSF is necessary and sufficient to express neural characteristics in MSCs and mediates the neural differentiation induced by cAMP-elevating compounds, forskolin and IBMX.

We demonstrate that cAMP-elevating compounds do not directly induce a neural phenotype but de-repress it through the down-regulation of NRSF. It is also noteworthy that FI appears to control NRSF expression indirectly as its gene expression levels are unchanged after 3 days of FI treatment (Fig. 7A), suggesting an involvement of a posttranslational regulatory mechanism in the downregulation of NRSF induced by FI. In agreement with this result, we observed an increase in protein expression of the E3 ubiquitin ligase B-Trcp, which is currently the only known post-translational regulator of NRSF expression^30^. Our results suggest that FI treatment is activating physiologically relevant molecular machinery to induce neural differentiation. Furthermore, FI’s ability to partially transdifferentiate MSCs across the mesoderm-ectoderm barrier relies on NRSF and further suggests that FI is partially reprogramming MSCs through modulation of transcription factors.

Studies of neural differentiation of MSCs have used various factors for induction, including soluble chemicals (BHA, B-ME), growth factors (bFGF), hormones (RA), and morphogens (BDNF)^11,13,17^. Despite a lack of consistency in the differentiation protocols, these studies were able to achieve induction of neural characteristics in MSCs. Most report expression of Tuj1 and other common neural markers, such as TH and NSE as indication of neural differentiation. Additionally, Tropel *et al*. used calcium release as a measure of neurotransmitter sensitivity and Zhang *et al*. were able to replicate this effect^10,11^. Interestingly, bFGF used by Tropel and, FI used by Zhang both independently induced MSCs to gain dopamine sensitivity. bFGF mainly utilizes MAPK signaling whereas FI induce elevation of intracellular cAMP. This may suggest that various differentiation protocols could be regulating a common program that controls the neural phenotype which we propose is de-repression of neural genes through downregulation of NRSF. This also suggests that MSCs have an intrinsic neural differentiation potential.

While our evidence suggests that FI specifically downregulates NRSF post-translationally, it is possible that other neural differentiation inducers downregulate NRSF through transcriptional processes. NRSF is regulated by the SMAD family of TFs, notably SMAD1/5/8^34^ and its promoter region contains two SMAD binding elements required for its expression. SMAD has been implicated in neural differentiation, and differentiation protocols used to generate neural cells from iPS or ESCs frequently use SMAD inhibitors to facilitate differentiation^35–37^. This suggests that the SMAD signaling pathway is important for NRSF dependent neural differentiation and suggests a common pathway that various inducers could act on to cause neural differentiation. To this end, the MAPK pathway is also important for neural differentiation. While MAPK signaling is somewhat ubiquitous and is generally associated with growth, it also engages in morphogen dependent differentiation. bFGF is a common inducer of neural differentiation in MSCs and neurotrophins such as NGF, BDNF, and NT-3–4 signal through the MAPK pathway through Trk receptors. Thus, not surprisingly SMADs activity can be inhibited upon phosphorylation by MAPK^36,38^. Therefore, it is plausible that neural inducers signal through MAPK kinase to affect NRSF expression via the SMAD signaling pathway.

In neural cells, expression of NRSF is regulated on several levels. At the transcriptional level, NRSF expression can be induced by SMAD proteins and TCF/Lef^34,39^. It is regulated at the protein level by the ubiquitin-protease system as it is a substrate of the E3-ubiquitin ligase, B-Trcp^30^. Finally, the cellular localization of NRSF is important for its function. Being a transcriptional repressor, NRSF functions maximally when it is nuclear and allowed access to DNA. Regulatory mechanisms that prevent nuclear import of NRSF are important for the homeostasis neural cells. Indeed, the huntingtin protein has been shown to sequester NRSF in the cytoplasm to permit expression of neuronal proteins, most notably, BDNF. Mutations in huntingtin that affect this binding show increased NRSF in the nucleus and repression of neuronal genes that contribute to the Huntingtin’s Disease pathophysiology^40,41^. Whether these mechanisms hold in MSCs needs to be more rigorously tested but could provide a possible explanation for why MSCs exhibit any neural competency.

While our results demonstrate that NRSF is critical for FI induced neural differentiation of MSCs, the molecular mechanism explaining FI-dependent downregulation is still incomplete. It is encouraging that FI caused increased expression of B-Trcp as this E3 ligase is a known regulator of NRSF and is important for neural differentiation in neural progenitor cells^30^. Future work is needed to determine if and how FI induced neural differentiation depends on B-Trcp activity.

We show that FI causes MSCs to gain sensitivity to dopamine and several markers of the dopaminergic neuronal subtype. Interestingly, although it is known that NRSF represses the tryptophan hydroxylase (*Tph*) gene important for serotonin synthesis^42^, neither FI nor NRSF silencing caused an expected increase in *Tph* expression (data not shown) suggesting that FI favors induction of MSCs towards the dopaminergic lineage and that *Tph* is dependent on other factors. The ability of FI to increase TH expression could be of clinical relevance to modulate dopamine production to treat pathologies caused by the lack of dopamine. This implicates a potential role for FI in controlling the dopaminergic phenotype associated with pathologies, including schizophrenia, Parkinson’s Disease, addiction, and depression. Our results suggest that FI could be a useful approach to modulate dopamine behavior in stem cells.

We previously observed that treatment of MSCs with FI temporarily induces a dramatic change in cell morphology resembling neuron-like structures^10^. However, this effect is transient lasting only 12–24 h. We observed no change in cell morphology during NRSF silencing over 72 h (data not shown) suggesting that the FI induced early morphology change does not involve NRSF. However, this does not preclude a possible role of NRSF in morphology changes on a longer time scale as Yang *et al*.^26^ showed that NRSF knockdown in MSCs over 14 days displayed significant morphology changes.

Transcription factors are key determinants of cell fate and artificially affecting their expression is well documented to transdifferentiate cells from lineage to lineage. While our study uses cAMP-elevating compounds, forskolin and IBMX, to induce differentiation of MSCs, the study underscores the importance of downregulating NRSF as the critical mechanism for induction. Our results suggest that chemical induction of neural differentiation is not due to off-target or non-specific effects, but is dependent on changes in transcription factors. This further supports the claim that MSCs can, to an extent, undergo neural transdifferentiation by downregulation of NRSF.

Given the role of chromatin remodeling in stem cell reprogramming and transdifferentiation^43–45^ our proposed mechanism gives clarity to the mechanism for neural differentiation of MSCs. NRSF represses gene expression through epigenetic effectors such as HDAC1/2, G9a, and MeCP2^27^ by creating a chromatin repressive environment. By downregulating NRSF, FI could be de-repressing a chromatin environment that is competent towards neural differentiation. From this perspective, FI-NRSF induced differentiation is not so much reliant on transient expression of neural genes, but is dependent on chromatin remodeling.

In conclusion, we show that FI induces neural-like differentiation of MSCs through NRSF and that downregulation of NRSF is necessary for induction of the neural phenotype in MSCs. Finally, we hypothesize that various neural induction protocols ultimately converge on downregulating NRSF to induce the neural phenotype.

## Supplementary information

Supplementary Figures and Tables

Video 1

Video 2

Video 3

Video 4

Video 5
