# Supplementary material for: Forskolin and IBMX Induce Neural Transdifferentiation of MSCs Through Downregulation of the NRSF
Source: Sci Rep. 2019 Feb 27;9:2969. doi: 10.1038/s41598-019-39544-0 (PMC6393535; doi:10.1038/s41598-019-39544-0)
Supplement: Supplementary file 1 — Supplementary Figures and Tables [file 41598_2019_39544_MOESM1_ESM.pdf]

**Forskolin and IBMX Induce Neural Transdifferentiation of MSCs Through Downregulation of the NRSF**

Ryan Thompson[1], Christina Casali[2], and Christina Chan[1, 2]

[1]Cell and Molecular Biology Program, Michigan State University, 567 Wilson Road, Rm 2240E, East Lansing, Michigan 48824, USA

[2] Department of Chemical Engineering and Materials Science, Michigan State University, 428 S. Shaw Lane, Rm 2527, East Lansing, Michigan 48824, USA

*Corresponding author

krischan@egr.msu.edu

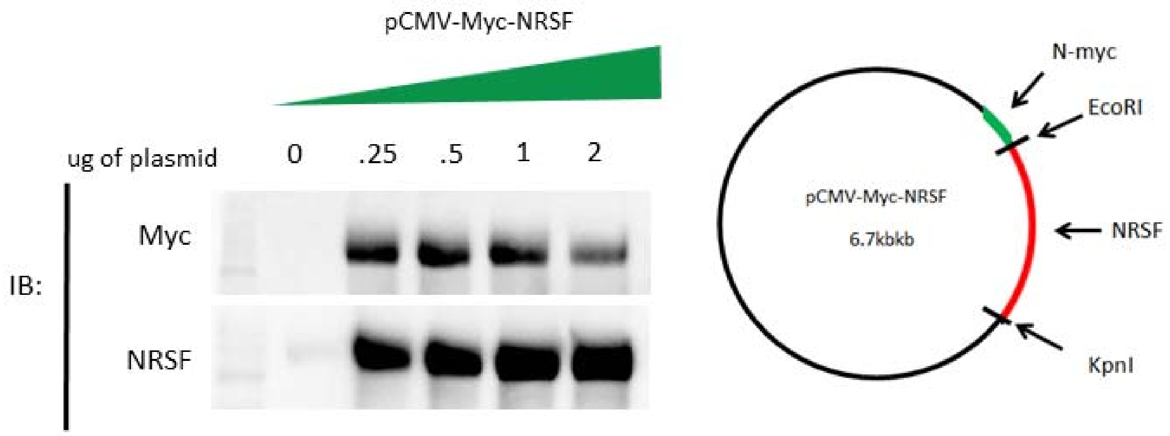


**Supplemental Figure 1. Overexpression of murine NRSF from pCMV-myc.**

Murine NRSF was cloned out of pHR'-NRSF-CITE-GFP and subcloned into pCMV-Myc-N from Clonetech. Transient overexpression shows an increase in signal of a myc product at ~200 kDa. An increase in immunoreactivity for NRSF was also observed at ~200 kDa.

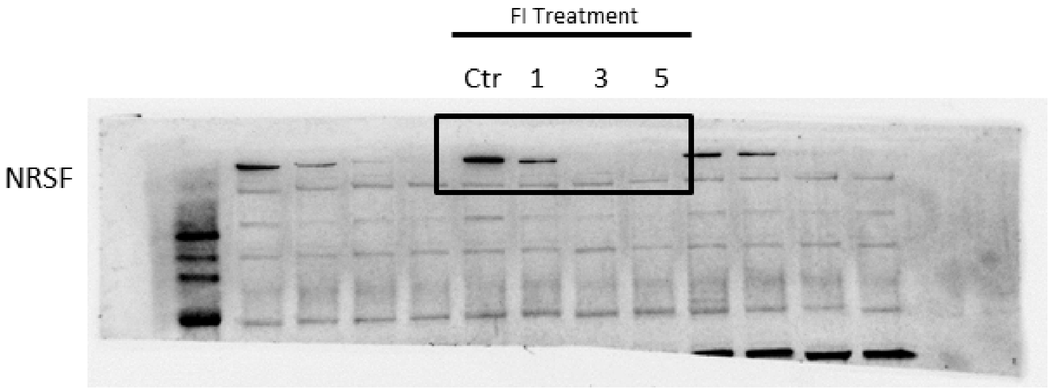


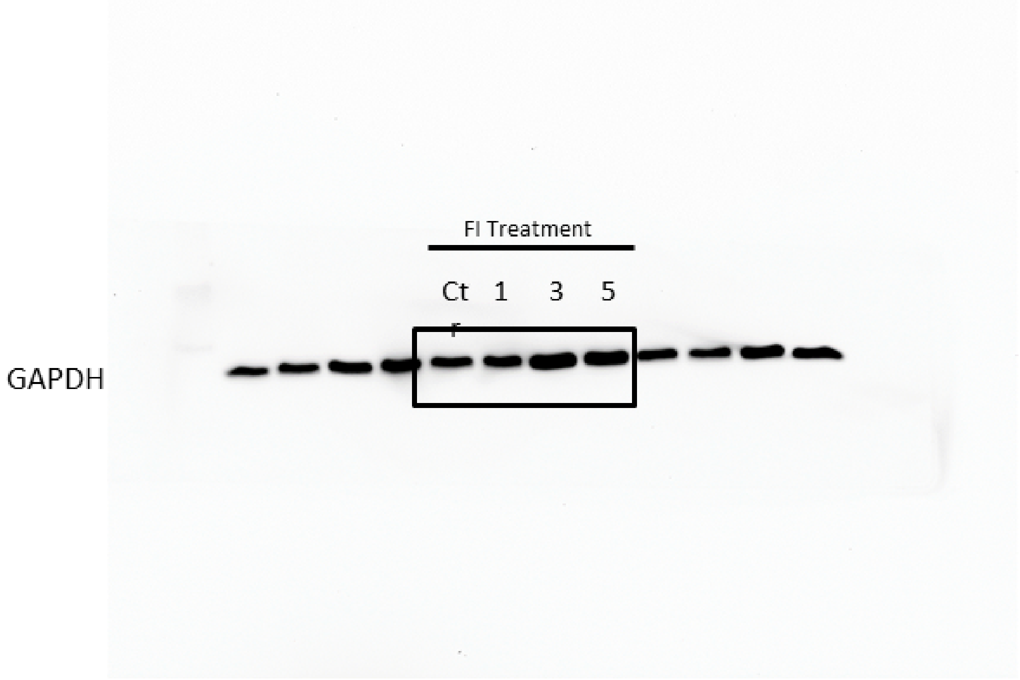


**Supplemental Figure 2. Full-length blots for Figure 1a.**

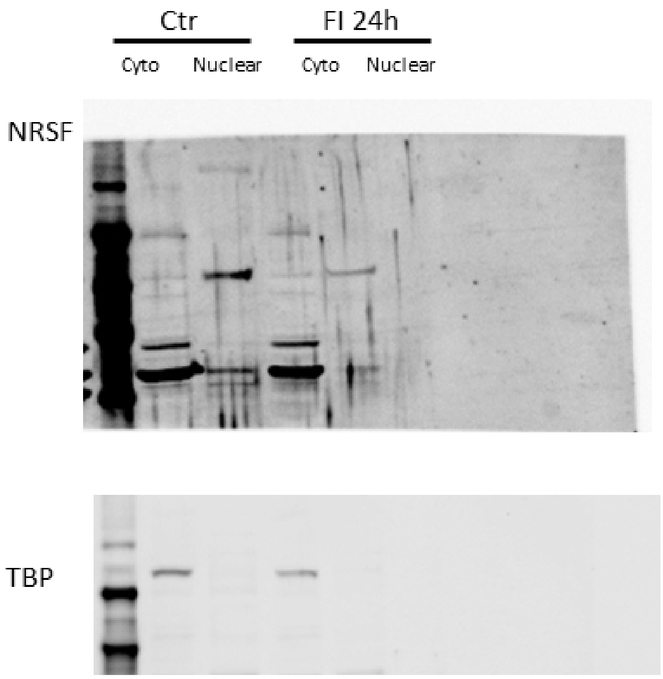


**Supplemental Figure 2. Full-length blots for Figure 1c.**

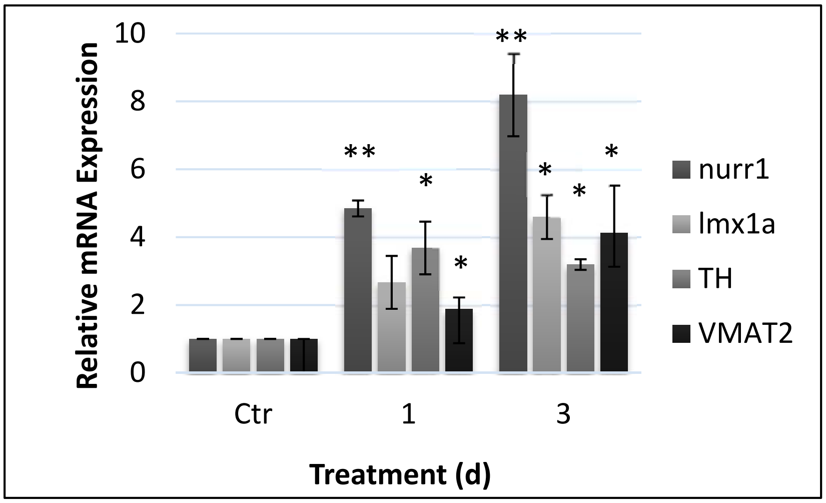


**Supplemental Fig 3. FI Treatment Induces Dopaminergic Genes in MSCs.**

Neural induction of MSCs with FI induces dopaminergic transcription factors nurr1 and lmx1a, and functional dopaminergic markers, TH and VMAT2. * $p < 0.05$; ** $p < 0.01$ as compared to control using Tukey's Test following ANOVA with N=3.

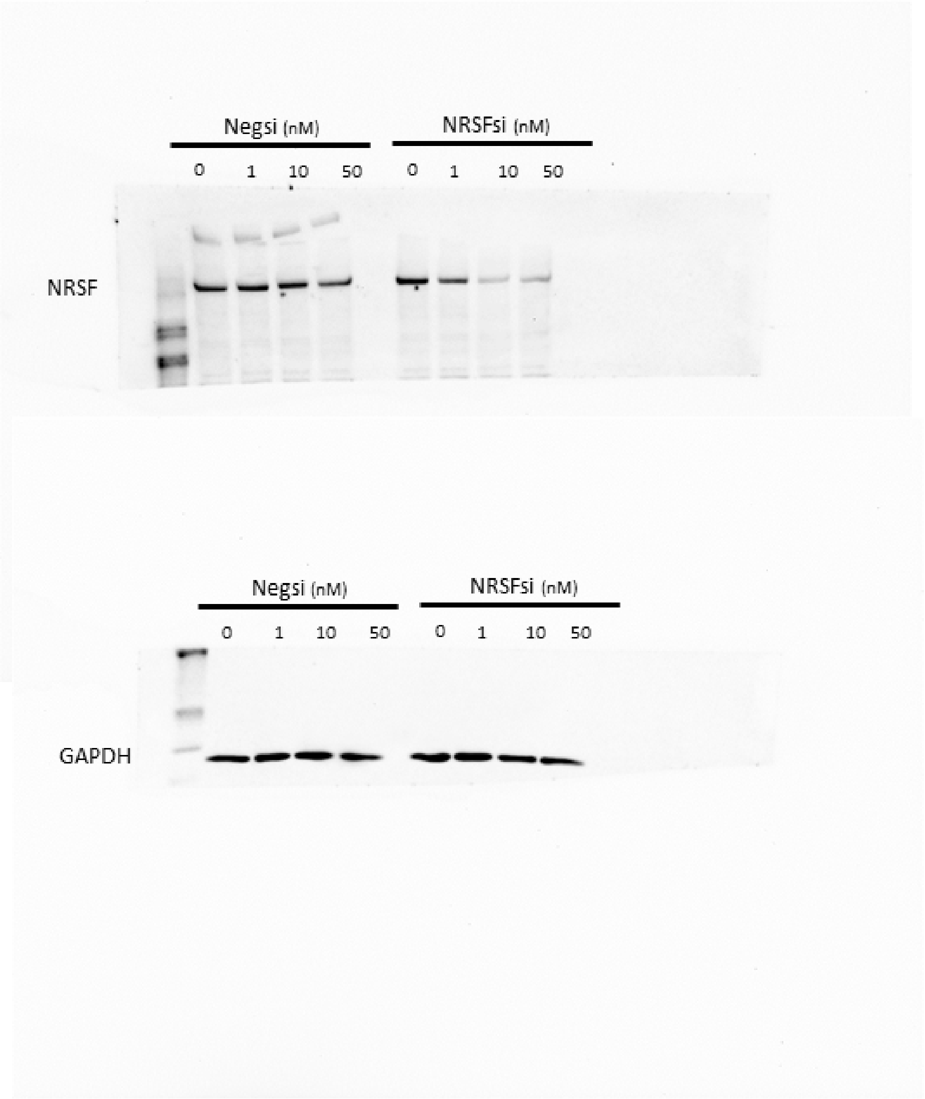


**Supplemental Figure 3. Full-length blots for Figure 3b.**

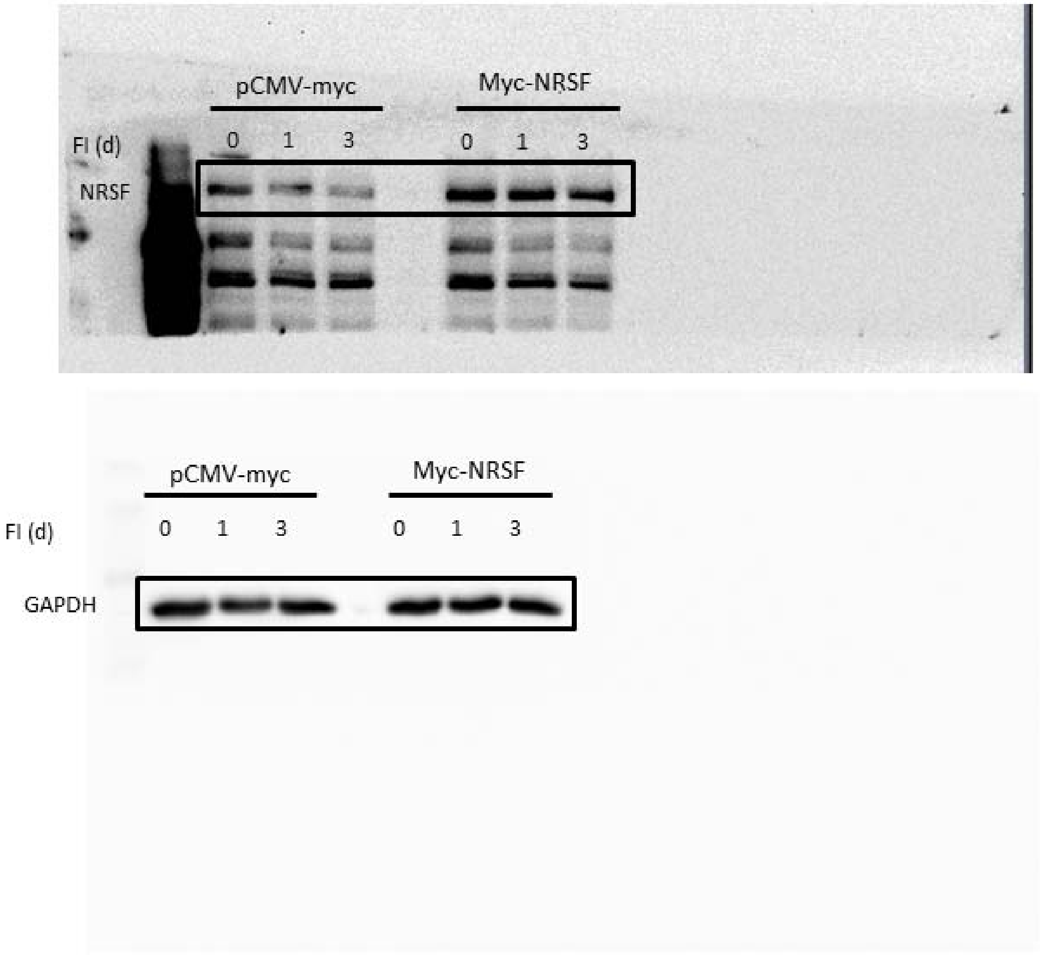


**Supplemental Figure 4. Full-length blots for Figure 4b.**

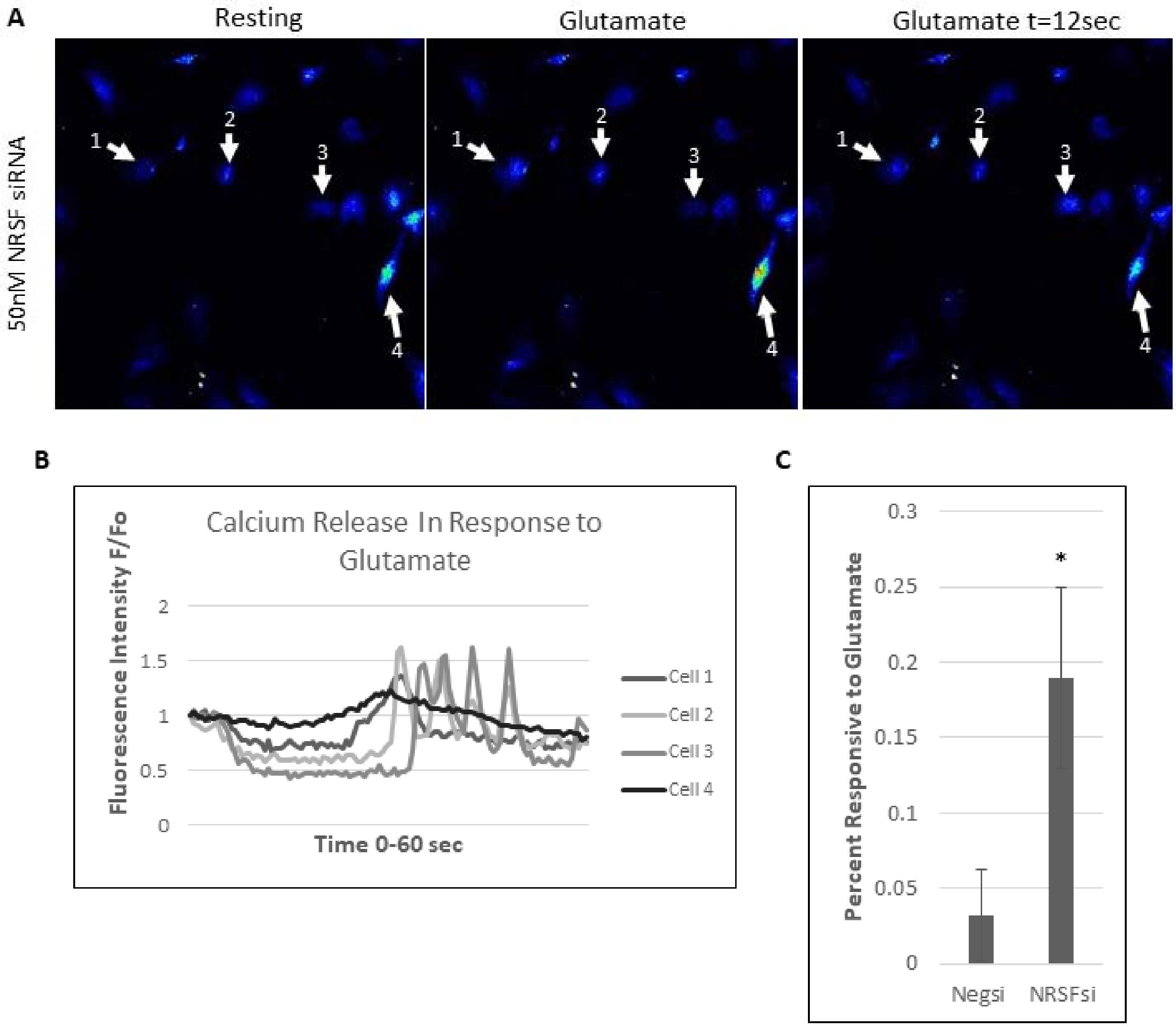


**Supplemental Figure 5. Knockdown of NRSF with siRNA Induces Glutamate Sensitivity in MSCs.**

A. Glutamate sensitivity in MSCs silenced with 50nM. A small portion of MSCs with NRSF expression knocked down exhibit calcium release when exposed to glutamate. B. F/Fo intensity plot for cells 1-4. Fluorescence intensity of select cells. Cells that show greater than 20% increase in fluo-4 fluorescence are counted as responsive. Images were collected over 60 sec. C. Quantification of cells responsive to glutamate exposure. For negsi N=3; For 50nM NRSFsi N=6. * $p > 0.05$ using students T-test.

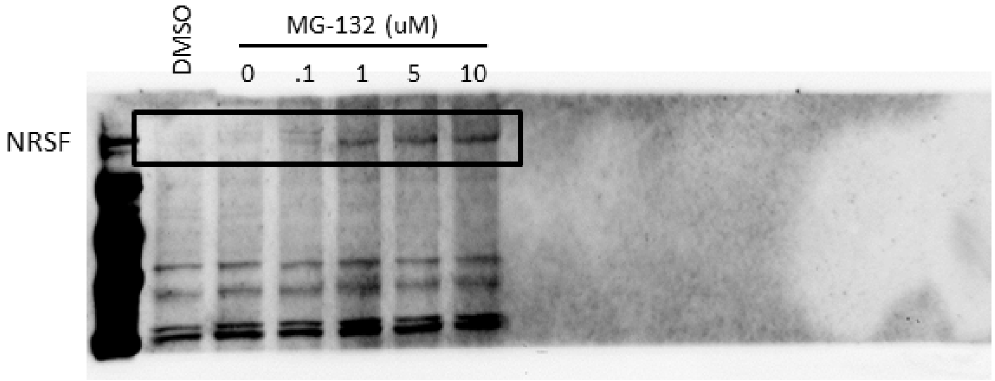


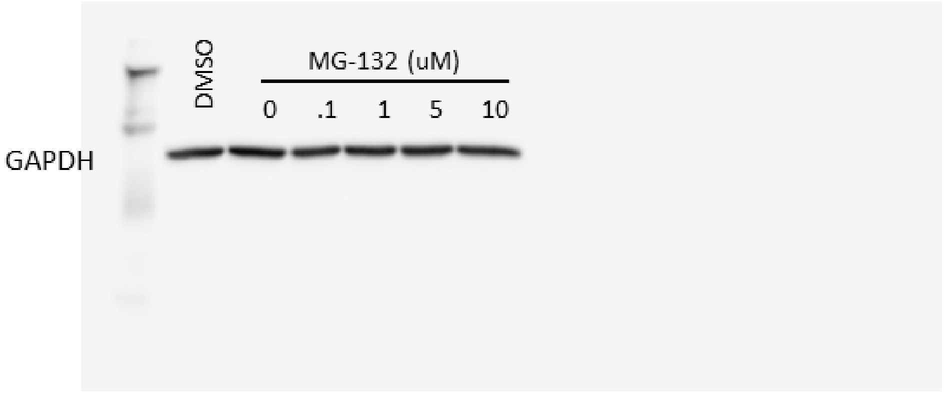


**Supplemental Figure 6. Full-length blots for Figure 7b.**

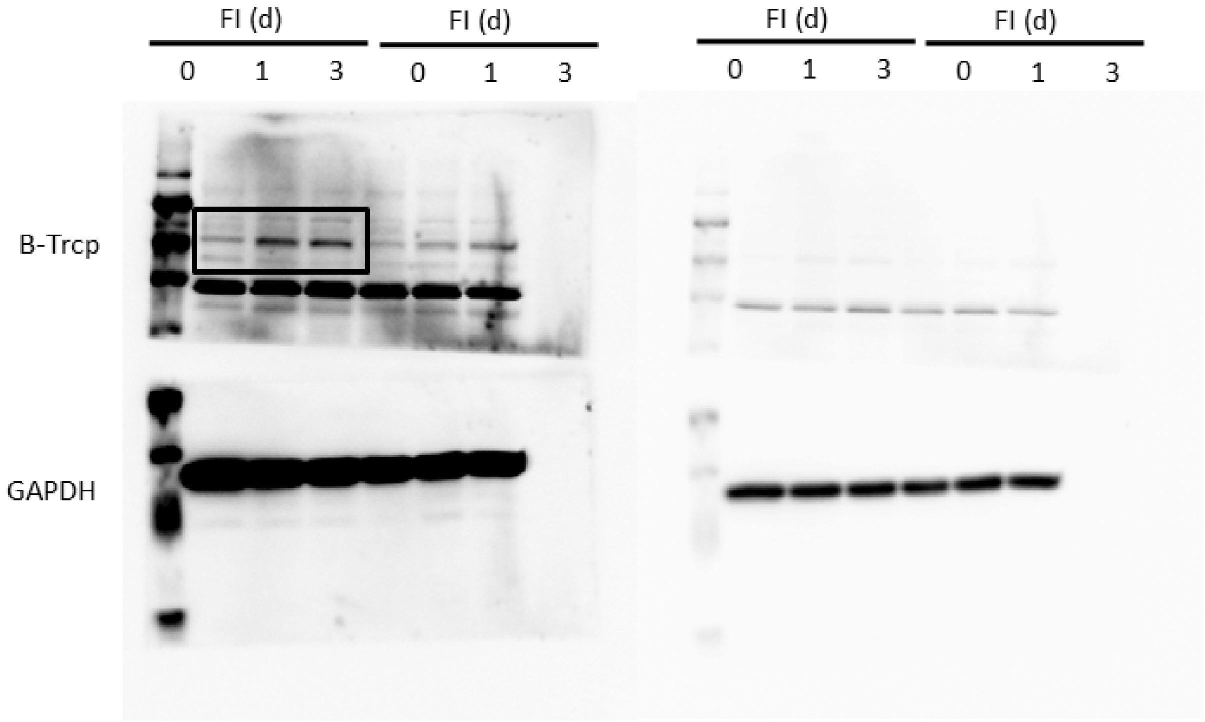


**Supplemental Figure 6. Full-length blots for Figure 7c.**

**Supplemental Table 1. Materials**

| Item | Company | Catalog Number |
|---|---|---|
| DMEM, low glucose | Gibco | 10567 |
| 70-uM nylon mesh | BD Falcon | 352350 |
| FBS | Gibco | 16000 |
| .25% Trypsin-edta | Gibco | 25200 |
| Forskolin | Sigma | F6886 |
| IBMX | Sigma | I5879 |
| Opti-MEM | Gibco | 31985 |
| Lipofectamine 3000 | Invitrogen | L3000 |
| Nitrocellulose | Biorad | 162-0112 |
| SuperSignal West Femto | Thermo | 34096 |
| Rneasy Mini Kit | Qiagen | 74104 |
| High Capacity cDNA RT Kit | Applied Biosystems | 4368814 |
| iQ Sybr Green Supermix | Biorad | 170-8882 |
| Chambered Cover-glass | Lab-Tek | 177429 |
| poly-L Lysine 70k-150k | Cultrex | 3438-200-01 |
| Fluo-4 | Invitrogen | F14201 |
| DAPI | Thermo Scientific | D3571 |

**Supplemental Table 2. Antibodies**

| Antibody | Dilution | Company | Catalog Number |
|---|---|---|---|
| NRSF | 1:2000 | Millipore | 07-579 |
| GAPDH | 1:10000 | Cell Signaling Technology | 2118 |
| TBP | 1:2000 | Cell Signaling Technology | 8515 |
| TH | 1:2000 | Cell Signaling Technology | 2792 |
| B-Trcp | 1:2000 | Invitrogen | 373400 |
| Goat-anti Rabbit | 1:10000 | Thermo Scientific | 31460 |
| Goat-anti Mouse | 1:2000 | Thermo Scientific | 31430 |

## Supplemental Table 3. Primer Sequences

| Primers (RT-PCR) | Forward | Reverse | Reference |
|---|---|---|---|
| NSE | TTGTTCTCAGTCCCATCCAA | ACCACATCAACAGCACCATC | Thompson 2018 |
| Tuj1 | CAGACACAAGGTGGTTGAGG | AGTGGAGAACACGGATGAGA | Thompson 2018 |
| TH | GCCCCACCTGGAGTATTTTGTGC | GCTCCCCATTCTGTTTACATAGCCCGAAT | Thompson 2018 |
| NRSF | AGCGAGTACCACTGGCGGAAACA | AATTAAGAGGTTTAGGCCCGTTG | Spencer 2006 |
| 18S | ACTCAACACGGGAAACCTCA | AATCGCTCCACCAACTAAGA | Ferreira 2012 |
| Drd1 | AAGCTTCTCAAACTCACTGATTCC | AACTCAACTCCTACCCTTCCTTTC | Thompson 2018 |
| Drd5 | TGCCTCCATCCTGAATCTGTGTA | GGATGAAGGAGATGAGGATGGA | Thompson 2018 |
| Nurr1 | CCTGACTATCAGATGAGTGG | CAGTTTGGACAGGTAGTTGG | Thompson 2018 |
| VMAT2 | CGGGACAGCCGCCACTCGCGCAAACT | GTGTCCCCTTCTGACTCTCTGGCTGT | Thompson 2018 |
| Lmx1a | ATTGCGCCCAATGAGTTTGT | GTGGGCAACGTTGTATAGGG | Thompson 2018 |
| | | | |
| **Primers (Cloning)** | **Forward** | **Reverse** | **Reference** |
| EcoRI-mNRSF | ACTGTAGAATTCAGGTTATGGCCACCAGGTGA | KpnI-mNRSF | Thompson 2018 |
| KpnI-mNRSF | EcoRI-mNRSF | CATGATGGTACCCTCAGCTACTCCTGCTCCT | Thompson 2018 |

**Supplemental Video 1. Dopamine Sensitivity of MSCs Negsi 50nM**

**Supplemental Video 2. Dopamine Sensitivity of MSCs NRSFsi 50nM**

**Supplemental Video 3. Dopamine Sensitivity of FI-Induced MSCs Overexpressing pCMV-Myc**

**Supplemental Video 4. Dopamine Sensitivity of FI-Induced MSCs Overexpressing pCMV-Myc-NRSF**

**Supplemental Video 5. Glutamate Sensitivity of MSCs NRSFsi 50nM**
